# A role for eIF5A in translational control of stress‐inducible proteins in multiple myeloma cell lines

**DOI:** 10.1002/1878-0261.70328

**Published:** 2026-10-21

**Authors:** Demi Both, Ingrid A. M. Derks, Raphaël Vanleyssem, Marine Leclercq, Najla El Hachem, Pierre Close, Eric Eldering, Marco Vincent Haselager

**Affiliations:** ^1^ Department of Experimental Immunology, Amsterdam UMC University of Amsterdam The Netherlands; ^2^ Lymphoma and Myeloma Center Amsterdam LYMMCARE Amsterdam The Netherlands; ^3^ Cancer Center Amsterdam Amsterdam The Netherlands; ^4^ Amsterdam Infection & Immunity Institute Amsterdam The Netherlands; ^5^ GIGA‐Institute Université de Liège Liège Belgium; ^6^ WELBIO Department WEL Research Institute Wavre Belgium

**Keywords:** apoptosis, eIF5A, ER stress, multiple myeloma, translation, UPR

## Abstract

Impaired protein folding in the endoplasmic reticulum (ER) causes ER stress and activates the unfolded protein response (UPR), which can induce apoptosis during prolonged ER stress. ER stress is therapeutically targeted in plasma cell malignancies, such as multiple myeloma (MM), but these treatments remain noncurative, and UPR signaling in B‐cell malignancies is incompletely understood. A targeted CRISPR‐Cas9 screen in RPMI‐8226 cells exposed to thapsigargin identified known ER stress regulators (XBP1, IRE1, and calreticulin) and the translation factor eIF5A (eukaryotic translation initiation factor 5A), whose role in ER stress remains unclear. Competition assays showed that eIF5A depletion is detrimental under basal conditions but confers a proliferation advantage during thapsigargin treatment. Inducible *EIF5A* knockouts confirmed reduced sensitivity specifically to thapsigargin, loss of eIF5A had no or sensitizing effects on cell death induced by other ER stress drugs. Polysome profiling revealed that not general translation, but the translation of specific mRNA transcripts coding for UPR components and apoptotic proteins was affected by eIF5A depletion. Correspondingly, *EIF5A* KO cells showed reduced thapsigargin‐induced UPR activation. In conclusion, these findings indicate that loss of eIF5A alters the stress response in MM cells.

## Graphical abstract

Loss of eIF5A rewires thapsigargin‐mediated endoplasmic reticulum stress responses in multiple myeloma cells by selective translation of mRNA transcripts, consequently dampening thapsigargin‐induced unfolded protein response (UPR) activation and shifting the balance from induction of apoptosis toward cell survival and proliferation.

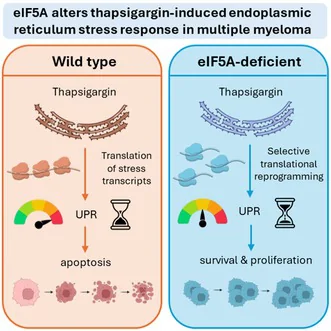

**Abbreviations**
BO: bortezomib
CHX: cycloheximide
dox: doxycycline
eIF: eukaryotic translation initiation factor
eIF5A: eukaryotic translation initiation factor 5A
ER: endoplasmic reticulum
GC7: N1‐guanyl‐1,7‐diaminoheptane
gRNA: guide RNA
KO: knockout
MFI: mean fluorescence intensity
MM: multiple myeloma
OPP: O‐propargyl‐puromycin
qVD: quinoline‐Val‐Asp‐difluorophenoxymethylketone
TBST: tris‐buffered saline containing 0.1% Tween‐20
TG: thapsigargin
UPR: unfolded protein response
UT: untreated
WT: wild‐type

## 1 Introduction

Proteostasis is a balanced and dynamic state in which the cellular proteome is continuously adjusted to meet specific physiological and environmental needs. A central component is protein production, through which genetic information is converted into functional proteins through transcription and translation [1]. Besides transcriptional control, protein production is also regulated at the level of mRNA translation. Translational control enables rapid and reversible modulation of protein synthesis in response to stress, developmental cues and metabolic demands, thereby maintaining cellular homeostasis [2]. Translation is a dynamic, multi‐step process comprising initiation, elongation and termination of polypeptide chains [3], and is carried out by ribosomes, tRNAs and numerous regulatory factors, such as eukaryotic translation initiation factor (eIF) proteins. Hence, translational regulation directly links protein production to proteostasis by regulating the production of correctly folded and functional proteins [1, 2, 3].

Proteins destined for secretion or the plasma membrane are produced following a specialized pathway [4]. These proteins are targeted to the endoplasmic reticulum (ER) and are typically synthesized by ribosomes bound to the ER membrane. Nascent polypeptides are co‐translationally translocated into the ER lumen or inserted into the plasma membrane [5]. Within the ER, they are further modified by disulfide formation and post‐translational modifications to ensure proper folding and functional proteins [6]. Conditions that impair protein folding will result in an accumulation of unfolded proteins in the ER lumen, a condition known as ER stress. To restore homeostasis, cells activate the unfolded protein response (UPR), which is a signaling network that transiently attenuates general translation but specifically enhances transcription of genes involved in protein folding, quality control and degradation [7]. The UPR is initiated by three different kinases PERK, IRE1, and ATF6, each activating distinct yet coordinated responses. PERK phosphorylates eIF2α to reduce general protein synthesis, while selectively inducing stress‐related genes, such as ATF4 and CHOP [8]. IRE1 catalyzes the splicing of XBP1 mRNA, thereby activating this transcription factor, which subsequently induces the expression of chaperones and components of ER‐associated degradation [9]. Upon activation, ATF6 is transported to the Golgi, where it is cleaved to release a transcription factor that similarly promotes chaperone expression [10]. When these adaptive mechanisms fail to resolve ER stress, prolonged UPR activation can shift toward cell death [11].

B lymphocytes, particularly antibody‐secreting plasma cells, are highly dependent on protein production in the ER due to their high production of antibodies [12]. Malignant B cells are even more vulnerable to ER stress, because of their increased proliferation and metabolic demands [13, 14, 15]. Pharmacological induction of ER stress by proteasome inhibitors such as bortezomib and carfilzomib is one of the therapeutic strategies for treating B‐cell malignancies, such as multiple myeloma (MM) [16, 17, 18]. However, current treatments remain noncurative, underscoring the need to delineate the exact wiring of UPR signaling in B‐cell malignancies to uncover therapeutically targetable vulnerabilities [19].

Thapsigargin is a commonly used ER stress inducer, by inhibiting Sarco(endo)plasmic Reticulum Calcium ATPase (SERCA) pump and subsequent depletion of calcium from the ER, which is needed for chaperone function [20]. Unlike proteasome inhibitors, which both induce ER stress and broadly disrupt protein degradation, thapsigargin specifically induces the UPR and can be used to study how its balance between cell repair and apoptotic signaling can be tipped toward cell death. In this study, we investigated ER stress and cell death pathways, to gain more insight in signaling pathways and potentially discover new targets for therapy.

Using a targeted CRISPR‐Cas9 screen, we identified eukaryotic translation initiation factor 5A (eIF5A) as a critical mediator of thapsigargin‐induced cell death. eIF5A is the only protein that contains the post‐translational modification hypusine, which makes it clinically interesting as therapeutic target [21, 22]. Depleting eIF5A from MM cells afforded rescue from thapsigargin‐induced cell death, most likely via inhibiting the synthesis of UPR proteins and, as part of a feedback mechanism, regulation of eIF5A activity. These results reveal a novel connection between the translational machinery and ER stress‐induced cell death in MM cells, with potential implications for therapeutic targeting.

## 2 Materials and methods

### 2.1 Cell culture

RPMI‐8226 cells (RRID:CVCL_0014; obtained from ATCC), LP‐1 (RRID:CVCL_0012; obtained from DSMZ) and JeKo‐1 (RRID:CVCL_1865; obtained from DSMZ) were cultured in IMDM (Thermo Fisher Scientific, Waltham, MA, USA) supplemented with 10% fetal bovine serum and 1% penicillin–streptomycin at 37 °C and 5% CO_2_. Cell lines were authenticated by short tandem repeat analysis and the absence of mycoplasma was routinely checked. To induce ER stress, the following drugs were used: thapsigargin (TG, Merck, Darmstadt, Germany), tunicamycin (Calbiochem, San Diego, CA, USA), brefeldin A (Merck), bortezomib (BO, Santa Cruz, Dallas, TX, USA). Hypusination of eIF5A was inhibited by the addition of N1‐guanyl‐1,7‐diaminoheptane (GC7, Calbiochem) [23, 24]. When indicated, cells were cultured in the presence of the pan‐caspase inhibitor QVD‐OPh (qVD, APExBIO, Houston, TX, USA).

### 2.2 Targeted CRISPR Cas9 screen

A targeted CRISPR‐Cas9 knockout (KO) screen was performed to identify genes involved in mediating thapsigargin‐induced cell death. A list of approximately 500 genes involved in processes such as ER stress, different cell death pathways and protein synthesis was designed (Table S1). Based on this list, a pooled guide RNA (gRNA) library, containing 4 gRNAs per gene chosen from the TKO library [25], cloned into lentiGuide‐Puro, a gift from Feng Zhang (Addgene plasmid no.: 52963; http://n2t.net/addgene:52963; RRID:Addgene_52 963 [26]), was produced by GenomeScan. RPMI‐8226 cells were first transduced with Lenti‐Cas9‐2A‐Blast, a gift from Jason Moffat (Addgene plasmid no.: 73310; http://n2t.net/addgene:73310; RRID:Addgene_73 310 [27]), and selected with Blasticidin (Merck). Afterward, the cells were transduced with the pooled library at a low multiplicity of infection (MOI < 0.3) to ensure single gRNA integration per cell. Transduced cells were selected with puromycin (Merck) for 48 h and maintained to preserve 1000× coverage of the library. Following selection, cells were cultured untreated (UR) or thapsigargin (RPMI‐8226 1 nm and JeKo‐1 2.5 nm) for 8–10 population doublings. Genomic DNA was extracted from cells directly after selection (T1) and after treatment conditions (T2), using the QIAamp DNA Blood Mini Kit (Qiagen, Venlo, The Netherlands). gRNA sequences were amplified by two‐step PCR and quantified by next‐generation sequencing (Illumina, San Diego, CA, USA). Differential gRNA representation was analyzed using the MAGeCK pipeline [28].

### 2.3 Generation of inducible CRISPR Cas9 KOs

Single‐gene KO cell lines were generated using an inducible CRISPR‐Cas9 system. RPMI‐8226, LP‐1 and JeKo‐1 cells stably expressing Cas9 were transduced with the lentiviral vector FgH1tUTG containing constitutive GFP expression and a doxycycline‐inducible gRNA cassette (Addgene plasmid no.: 70183; http://n2t.net/addgene:70183; RRID:Addgene_70 183 [29]). For *EIF5A, DHPS*, and *DOHH*, three different gRNAs per gene were cloned separately and compared with a gRNA targeting the AAV1S locus as a control (mock). Transduced cells were kept in culture, and KOs were induced by adding doxycycline (dox, 1 μg·mL^−1^) 4 (*EIF5A*) or 8 (*DHPS* and *DOHH*) days prior to experiments. KO efficiency was verified by western blot. A list of gRNA sequences is provided in Table S2.

### 2.4 Western blot

Cells were lysed in ice‐cold RIPA buffer (Thermo Scientific, Waltham, MA, USA) supplemented with protease and phosphatase inhibitors (Roche). Lysates were incubated on ice for 30 min and cleared by centrifugation at 15 000 ***g*** for 10 min at 4 °C. Protein quantification was done by BCA protein Assay (Pierce, Stockbridge, GA, USA). Equal amounts of protein were mixed with Laemmli sample buffer and loaded on 13% gels and transferred onto PVDF‐FL membranes (Millipore, Burlington, MA, USA) for 1 h at 300 mA or overnight at 30 V. Membranes were then blocked for 1 h at room temperature (RT) in 2% nonfat dry milk or 5% BSA in Tris‐buffered saline containing 0.1% Tween‐20 (TBST), then incubated overnight with primary antibodies diluted in 1% milk solution. After three washes with TBST, membranes were incubated with IRDye labeled secondary antibodies for 2 h at RT. After three washes, membranes were dried and scanned either on Odyssey Imager (Li‐Cor Biosciences, Lincoln, NE, USA) or ChemiDoc MP imaging system (Bio‐Rad, Hercules, CA, USA). Quantification of bands was performed using imagej software (Version 1.50i). Protein levels were normalized to β‐actin as loading control. A list of antibodies is provided in Table S3.

### 2.5 Quantitative PCR

Total RNA was extracted from cells using the RNeasy Mini Kit (Qiagen) according to the manufacturer's instructions. RNA concentration and purity were assed using a NanoDrop spectrophotometer (Thermo Fisher Scientific) and total RNA was reverse‐transcribed into cDNA using RevertAid H Minus M‐MuLV Reverse Transcriptase (Fermentas, Waltham, MA, USA) and random hexamers (Promega, Madison, WI, USA). Quantitative PCR (qPCR) was performed with the QuantStudio 3 (Applied Biosystems, Waltham, MA, USA) using Fast SYBR Green Master Mix (Applied Biosystems) and standard settings. Data analysis was performed using LinReg PCR [30]. Relative gene expression was calculated using the delta–delta *C*_t_ (ΔΔ*C*_t_) method with TBP1 as housekeeping gene. Fold changes were calculated relative to untreated controls. A list of qPCR primers is provided in Table S4.

### 2.6 Flow cytometry

Cell death was assessed by flow cytometry using DiOC6 (Invitrogen) to measure mitochondrial membrane potential and TO‐PRO‐3 iodide (Invitrogen) to detect loss of plasma membrane integrity. Cells were plated in 96‐round bottom plates. A mix of DiOC6 and TO‐PRO‐3 was added and cells were incubated for 25 min at 37 °C in the dark. Samples were measured on BD FACS Canto or Celesta cytometers. At least 10 000 events per sample were recorded. Data were analyzed using the flowjo V10 software (FlowJo, LLC, Ashland, OR, USA). Viable cells were defined as DiOC6^+^/TO‐PRO‐3^−^. Specific cell death was calculated as (%cell death in treated sample − % cell death target cells in untreated sample)/(100 − % cell death cells in untreated sample) * 100%.

### 2.7 Competition assay

Cell competition assays were performed to assess relative fitness and survival of wild‐type (WT) and *EIF5A* KO cells under various conditions. *EIF5A* and mock single KOs were GFP‐labeled based on the earlier described inducible KO system and compared with unlabeled WT cells. WT and GFP‐labeled mock or *EIF5A* KO cells were mixed in a 1 : 1 ratio and co‐cultured for the indicated amount of time and KOs were induced in the first 4 days with doxycycline. When indicated, cells were treated with 5 μm qVD or the ER stress inducer thapsigargin (RPMI‐8226: 1 nm, LP‐1: 1.5 nm and JeKo‐1: 2.5 nm). GFP‐positive and negative populations were quantified by flow cytometry to determine the relative abundance of WT and *EIF5A* KO cells. Data were normalized to initial input.

### 2.8 Click‐iT OPP assay

Nascent protein synthesis was quantified using the Click‐iT Plus OPP Protein Synthesis Assay Kit (Thermo Fisher Scientific) according to manufacturer's instructions. In short, RPMI‐8226 mock and *EIF5A* KO cells were induced by addition of doxycycline as described before. After KO induction, cells were plated in 6‐well plates and treated with thapsigargin (6 nm) for 24 h. Cells were harvested, and incubated with 20 μm O‐propargyl‐puromycin (OPP) for 30 min at 37 °C to allow incorporation of OPP into newly synthesized polypeptides. As a positive control, cells were pretreated with 100 μg·mL^−1^ cycloheximide (CHX) (Merck) for 5 min at 37 °C before incubation with OPP. Following incubation, cells were washed twice with PBS and fixed and permeabilized by Cytofix/Cytoperm fixation and permeabilization kit (BD Biosciences, Franklin Lakes, NJ, USA) for 15 min at 4 °C. Fixed cells were subjected to the Click‐iT reaction using the Alexa Fluor 488 azide reagent to covalently label OPP‐containing proteins for 30 min at RT. After washing, cells were stained with Fixable Viability Dye eFluor™ 780 (eBioscience, San Diego, CA, USA), resuspended in PBA (PBS, 0.5% BSA and 0.02% Sodium Azide) and measured on a BD Celesta cytometer. Mean fluorescence intensity (MFI) of OPP‐Alexa Fluor 488 was quantified using flowjo software in which fluorescence of CHX‐treated samples was subtracted from all experimental values and OPP signal was calculated relative to untreated WT samples.

### 2.9 Polysome profiling and RNA seq

Polysome profiling was performed to assess translational activity in RPMI‐8226 mock and *EIF5A* KO cells. First, KOs were induced as described before and subsequently treated with 6 nm thapsigargin for 24 h. Cells were harvested, resuspended in ice‐cold PBS containing 100 μg·mL^−1^ CHX, centrifuged at 200 ***g*** and pellets were resuspended in hypotonic buffer (5 mm Tris–HCl (pH 7.5), 2.5 mm MgCl_2_, 1.5 mm KCl and 1× protease inhibitor cocktail (EDTA‐free)) and subsequent addition of CHX (100 μg·mL^−1^), 1 m DTT, RNAse inhibitor (Thermo Fisher Scientific), 10% Triton X‐100 (0.5%), and 10% Sodium Deoxycholate (0.5%). Optical densities (ODs) of cleared lysates were measured by Nanodrop at 260 nm and equalized by addition of hypotonic buffer. Equal amounts of clarified lysates (10–20 OD) were layered onto 5‐34%‐55% nonlinear sucrose gradients prepared in polysome gradient buffer and centrifuged at 222228 ***g*** for 4 h at 4 °C using SW60 rotor optima XPN‐80 or OPTIMA L‐90K ultracentrifuge. Gradients were fractionated using a Gradient Station (BioComp) with continuous monitoring of absorbance to visualize 40S, 60S, and 80S polysome peaks. Fractions were then collected for RNA isolation using TRIzol LS Reagent according to manufacturer's instructions. RNA from polysome fractions and input samples was quantified and checked for quality. From each sample, total RNA and polysome RNA were sent for RNA sequencing. The mRNA library preparation was performed using the TruSeq Stranded mRNA kit (20020595; Illumina). Sequencing was performed using the NovaSeq S1 (100 cycles) standard workflow.

### 2.10 Statistical analysis

*P*‐values were calculated using two‐sided paired or unpaired *t*‐tests or one‐way ANOVA (followed by Tukey's *post hoc* test). Statistical analysis was performed using graphpad prism (GraphPad, Boston, MA, USA) version 9.1.0 with significance set at *P < 0.05, **P < 0.01; ***P < 0.001. All statistical information is specified in the figure legends.

## 3 Results

### 3.1 Depletion of eIF5A protects specifically to thapsigargin‐induced cell death

To identify genes essential for the thapsigargin‐induced ER stress response in RPMI‐8226 cells, we performed a targeted CRISPR‐Cas9 screen (Fig. 1A). RPMI‐8226 cells were cultured for 10 days in the presence or absence of thapsigargin to identify genes that provide a (dis)advantage to thapsigargin treatment based on the under/overrepresentation of specific gRNAs (Fig. 1B). We looked for gRNAs that were enriched in the treated sample in order to find gene KOs that result in enhanced survival or proliferation under thapsigargin treatment (Fig. 1C). Among the top hits were *XBP1, ERN1* (IRE1), *CALR* (calreticulin), and *EIF5A* (eIF5A). For XBP1, IRE1, and calreticulin, their role in ER stress in MM is well known and thus confirmed the validity of the screen [14, 31]. The translation factor eIF5A has not yet been well characterized in the context of ER stress. Analysis of DepMap data indicated that *EIF5A* is an essential gene [32], with KO expected to result in cell death, particularly in hematopoietic and B‐cell malignancies. To validate this hit, we used an inducible system to generate single KO cell lines using the three individual *EIF5A* gRNAs used in the CRISPR‐Cas9 screen, each targeting different exons in the *EIF5A* gene and compared this to AAV1S‐transduced mock cells (Fig. S1A,B). Afterward, the best clone was selected. After induction with doxycycline, eIF5A protein levels were decreased by 50%, which did not affect basal viability (Fig. 1D, Fig. S1C). Loss of eIF5A specifically conferred increased resistance to thapsigargin‐induced cell death, while resulting in sensitization to bortezomib and brefeldin A, and had no effect on tunicamycin‐induced cell death (Fig. 1E). Notably, a second CRISPR‐Cas9 screen performed in thapsigargin‐treated JeKo‐1 cells, also showed enrichment of *EIF5A* KO cells in the treatment arm (Fig. S2A,B), although not statistically significant. Further comparison of hits in RPMI‐8226 and JeKo‐1 cells revealed both overlapping and distinct hits (Fig. S2C,D). These results indicate that eIF5A plays a role in mediating thapsigargin‐induced cell death in RPMI8226 cells.

**Fig. 1:**
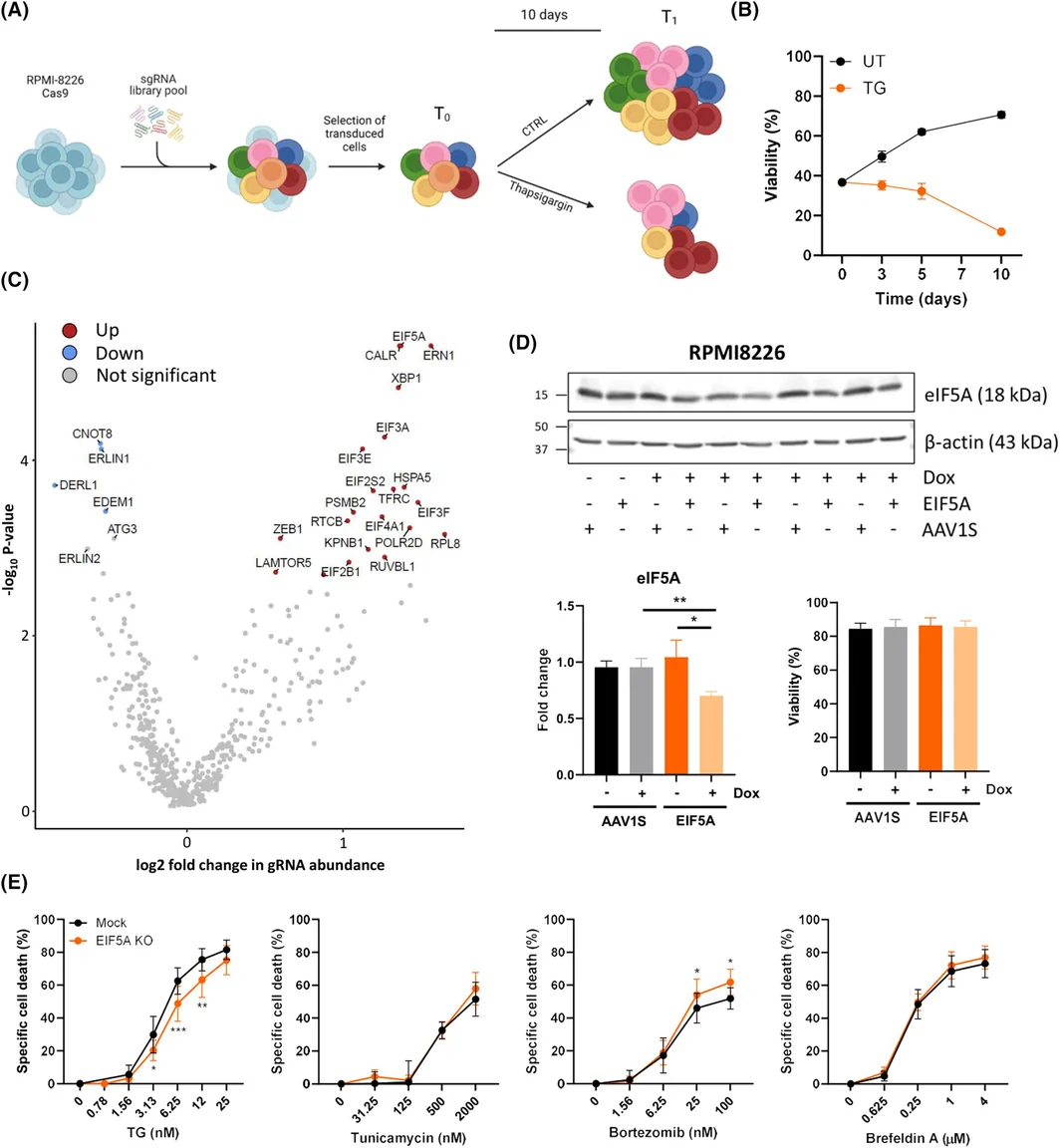
CRISPR‐Cas9 screen and single *EIF5A* knockout (KO) characterization. (A) Schematic overview of targeted CRISPR screen setup. (B) Viability of CRISPR‐Cas9 screen treatment arms after 10 days untreated (UT) or with thapsigargin (TG, 1 nm), *N* = 3. Data are presented as mean ± SD. (C) Volcano plot of guide RNA (gRNA) abundance from CRISPR‐Cas9 screen in UT vs TG arm. Enriched gRNAs (colored in red) and depleted gRNAs (colored in blue) are based on a *P*‐value threshold set at 0.05 and calculated using MAGeCK robust rank aggregation. (D) Western blot of three inducible single *EIF5A* KO clones treated with and without doxycycline (dox, 1 μg·mL^−1^) for 3 days with corresponding eIF5A quantification and viability, *N* = 5–8. Data are presented as mean ± SEM. *P*‐values were calculated using paired *t*‐tests, *P < 0.05, **P < 0.01. (E) Specific cell death of RPMI‐8226 mock and *EIF5A* KO cells treated with ER stress‐inducing drugs as indicated for 24 h, *N* = 4. Data are presented as mean ± SD. *P*‐values were calculated using or two‐way ANOVA tests, *P < 0.05, **P < 0.01, ***P < 0.001.

### 3.2 Loss of eIF5A is beneficial in thapsigargin‐induced ER stress conditions

To further investigate the protective effect of eIF5A loss upon thapsigargin treatment, we performed competition assays comparing *EIF5A* KO cells with WT cells in multiple cell lines, including RPMI‐8226 (Fig. 2A), LP‐1 (Fig. 2B) and JeKo‐1 (Fig. 2C) cells. Control experiments with GFP‐labeled mock‐transduced cells versus unlabeled WT cells showed that transduction with the mock plasmid did not change proliferation in all three cell lines compared with WT cells, which could then further be used to test the effect of *EIF5A* KO. When *EIF5A* KO cells were mixed with WT cells, *EIF5A* KO cells were outcompeted under basal culture conditions in all three cell lines, confirming the essentiality of the gene for cell growth. Addition of qVD did not rescue loss of KO cells (Fig. 2A, panel 3), suggesting this effect is not due to caspase‐mediated cell death. Interestingly, under thapsigargin‐induced ER stress, the growth disadvantage in KO cells was reversed, and eIF5A loss conferred a clear advantage over WT cells in all three cell lines (Fig. 2B,C), in alignment with the CRISPR‐Cas9 screen. These results demonstrate that eIF5A is generally required for cell growth but its absence becomes protective upon thapsigargin treatment.

**Fig. 2:**
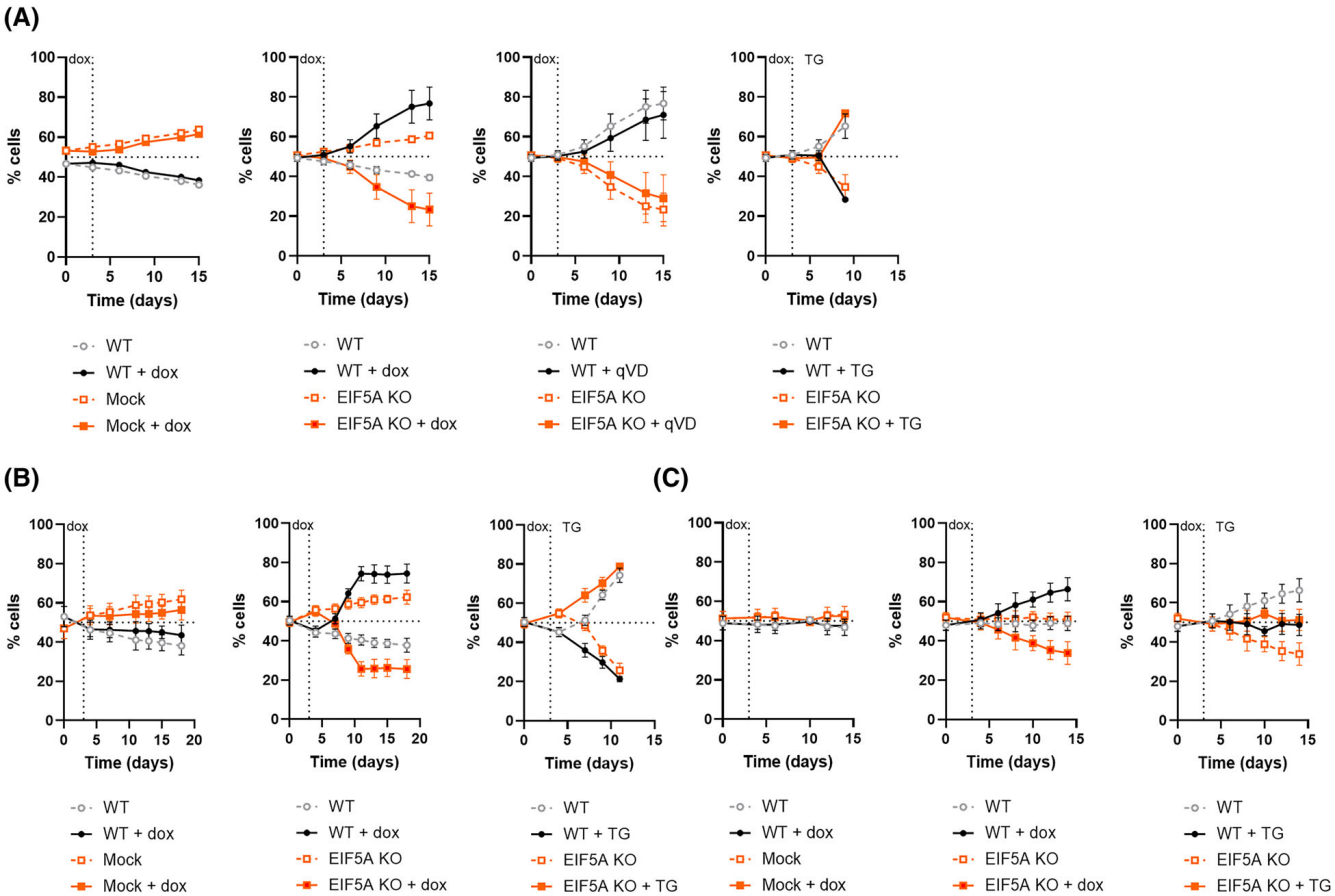
Relative fitness of mock vs *EIF5A* knockout (KO) cells in RPMI‐8226, LP‐1, and JeKo‐1 cells. (A) Competition assay in RPMI‐8226 wild‐type (WT) vs mock or vs *EIF5A* KO cells under basal conditions, with doxycycline (3 days, 1 μg·mL^−1^), qVD (5 μm) or TG (1 nm), *N* = 3. Data are presented as mean ± SEM. (B) Competition assay in LP‐1 WT vs mock or vs *EIF5A* KO cells under basal conditions, with doxycycline (3 days, 1 μg·mL^−1^ nm) or TG (1.5 nm), *N* = 4. Data are presented as mean ± SD. (C) Competition assay in JeKo‐1 WT vs mock or vs *EIF5A* KO cells under basal conditions, with doxycycline (3 days, 1 μg·mL^−1^ nm) or TG (2.5 nm), *N* = 4. Data are presented as mean ± SD.

### 3.3 eIF5A depletion affects translational regulation of stress transcripts

To investigate how eIF5A affects protein synthesis in the context of ER stress, we analyzed translation levels in mock and *EIF5A* KO cells in the absence and presence of thapsigargin. Translation was measured using OPP, a puromycin analog that incorporates into nascent polypeptides, and is subsequently detected by a click reaction resulting in fluorescence. Whereas thapsigargin treatment significantly inhibited OPP incorporation in mock cells, loss of eIF5A resulted in a nonsignificant reduction in OPP signal, suggesting a minor effect of eIF5A on general translation levels (Fig. 3A). Correspondingly, translation efficiency as determined by polysome profiles did not show differences between mock and eIF5A‐depleted cells (Fig. 3B). We found that a large proportion of polysome‐associated transcripts that were actively translated in mock cells were lost in *EIF5A* KO cells upon treatment with thapsigargin (Fig. 3C). This group included many transcripts related to protein homeostasis, such as proteasome subunits, chaperones, stress sensors, translation factors, and many related to cell cycle progression and proliferation (Fig. 3D). On the contrary, transcripts coding for the calcium‐dependent chaperone calreticulin (CALR) were uniquely translated in *EIF5A* KO cells and absent in the polysomal fraction in mock cells treated with thapsigargin. These differences suggest a specific role for eIF5A in translation upon thapsigargin treatment rather than global translation. Polysome profiling allowed investigation of different modes of translational regulation, by categorizing transcripts into three groups based on the ratio of polysomal‐associated transcripts to total RNA transcripts. First, translation refers to changes in polysome‐associated mRNA independent of total mRNA levels (translation efficiency), buffering describes stable polysome‐associated mRNA independent of total mRNA (to maintain stable protein translation), and abundance reflects proportional changes in both total and polysomal fractions (no translational regulation). Whereas mock cells treated with thapsigargin had a large portion of transcripts assigned to the translation category, *EIF5A* KO cells had more transcripts in the buffering and abundance categories upon treatment (Fig. 3E,F), suggesting different modes of translational regulation upon thapsigargin treatment. The most significant changes were transcripts shifting from the translation category in mock cells to abundance in *EIF5A* KO cells and from abundance in mock cells to translation and buffering categories in *EIF5A* KO cells (Fig. 3G). Among these transcripts were multiple key UPR effectors (Fig. 3H). For instance, in *EIF5A* KO cells, stress‐responsive transcripts such as PERK (*EIF2AK3*) and CHOP (*DDIT3*) were no longer translationally regulated upon thapsigargin treatment, whereas transcripts coding for chaperones, including BiP (HSPA5) and DNAJC1 shifted toward regulation at the translational level, and BIRC5 transcripts were found only in the buffering category in *EIF5A* KO cells. Since eIF5A may rescue ribosomes from stalling when encountering stretches of consecutive proline residues [33], we investigated enrichment of polyproline stretches within transcripts across the different polysomal categories in mock and EIF5A KO cells using analysis of motif enrichment [34]. We observed a significant enrichment of (Pro)5–6 stretches in buffered transcripts uniquely in EIF5A KO cells, suggesting ribosome accumulation on polyproline stretches leading to mRNA degradation in EIF5A KO cells (Fig. 3I). Furthermore, transcripts within the translation category revealed a significant enrichment of (Pro)17 stretches in mock cells, while no such enrichment was found in EIF5A KO cells, suggesting that translation of transcripts containing longer polyproline stretches is abrogated upon depletion of eIF5A. Together these results indicate that eIF5A loss did not significantly alter total protein synthesis levels under thapsigargin‐induced stress, yet selectively reshaped the translation of stress‐related transcripts, potentially leading to a fine‐tuned and distinct adaptive response to thapsigargin‐induced ER stress.

**Fig. 3:**
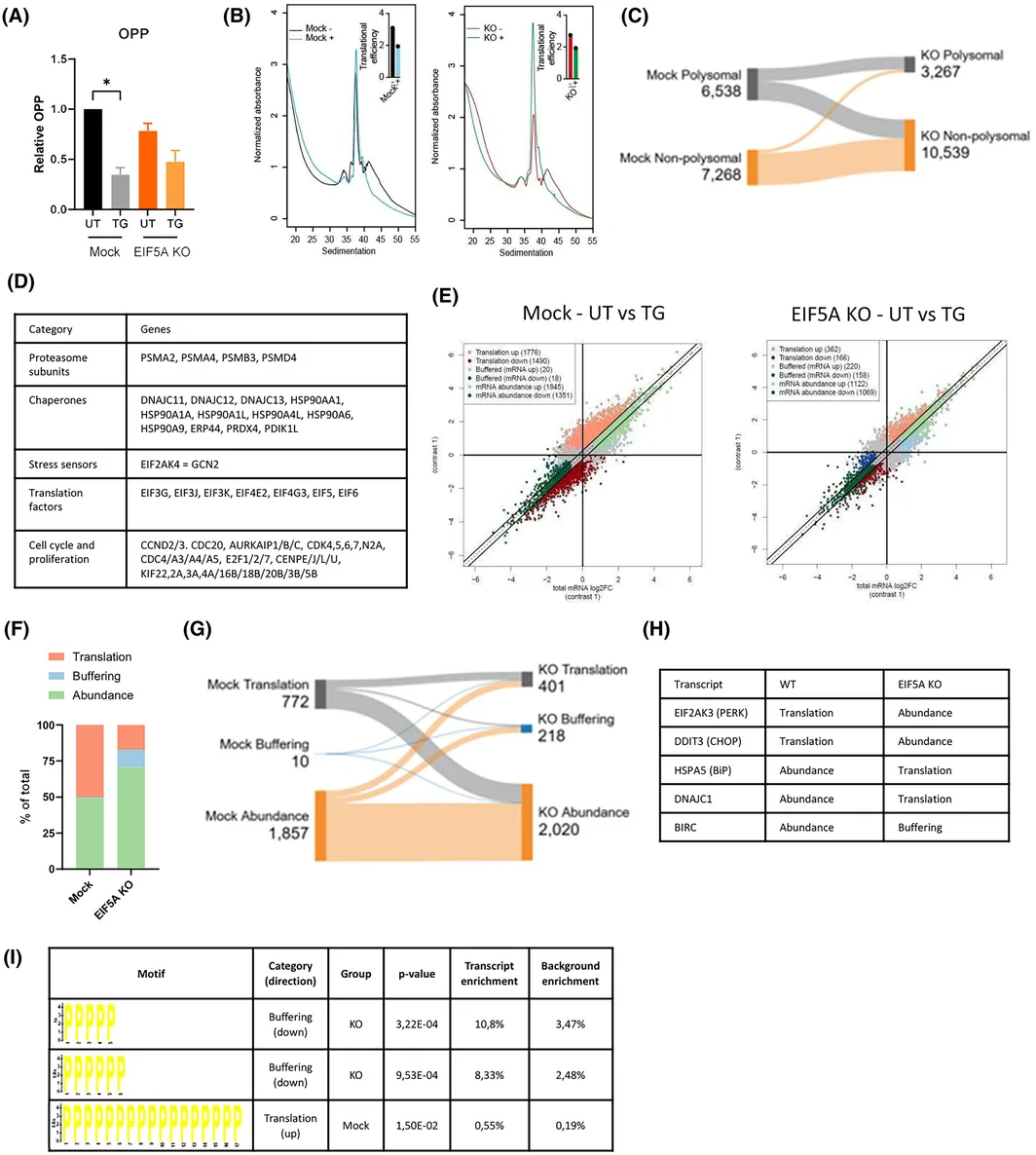
Effect of *EIF5A* knockout (KO) on translation upon thapsigargin. (A) O‐propargyl‐puromycin (OPP) assay in RPMI‐8226 mock vs *EIF5A* KO cells after TG (24 h, 6 nm), *N* = 3. Data are presented as mean ± SEM. *P*‐values were calculated using paired *t*‐tests, *P < 0.05. (B) Polysome profiles of RPMI‐8226 mock and *EIF5A* KO with TG (24 h, 6 nm), *N* = 4, one representative experiment is shown. (C) Sankey plot of polysomal vs nonpolysomal fractions in mock and *EIF5A* KO after TG (24 h, 6 nm). (D) Selection of transcripts in polysomal fraction in mock cells that shift to the nonpolysomal fraction in *EIF5A* KO cells. (E) Distribution of number of transcripts across categories in polysomal fractions in mock and *EIF5A* KO upon TG (24 h, 6 nm). (F) Quantification of E. (G) Sankey plot of polysomal transcripts across different categories in mock and *EIF5A* KO. (H) Selection of transcripts shifting categories between mock or *EIF5A* KO. (I) Results of polyproline motif enrichment analysis. TG, thapsigargin.

### 3.4 Loss of eIF5A attenuates UPR induction upon thapsigargin treatment

To investigate whether eIF5A depletion affects UPR induction upon thapsigargin, we measured gene expression and protein levels of targets involved in the UPR. Consistent with the polysome profiling data, *EIF5A* KO cells showed a muted induction of several UPR effectors upon thapsigargin treatment at the mRNA level (Fig. 4A,B, Fig. S3). This reduction was most pronounced for the PERK arm of the UPR, including PERK (*EIF2AK3*) and eIF2α (*EIF2S1*). At the protein level, the attenuated upregulation of UPR effectors in the KO cells mirrored the transcriptional pattern, showing significant reduction of PERK and CHOP in *EIF5A* KO cells upon thapsigargin treatment (Fig. 4C,D). Furthermore, loss of eIF5A did not significantly affect expression of pro‐ and anti‐apoptotic proteins during thapsigargin‐induced ER stress (Fig. S4A–C). Together, these results indicate that eIF5A depletion blunts canonical UPR activation upon thapsigargin treatment.

**Fig. 4:**
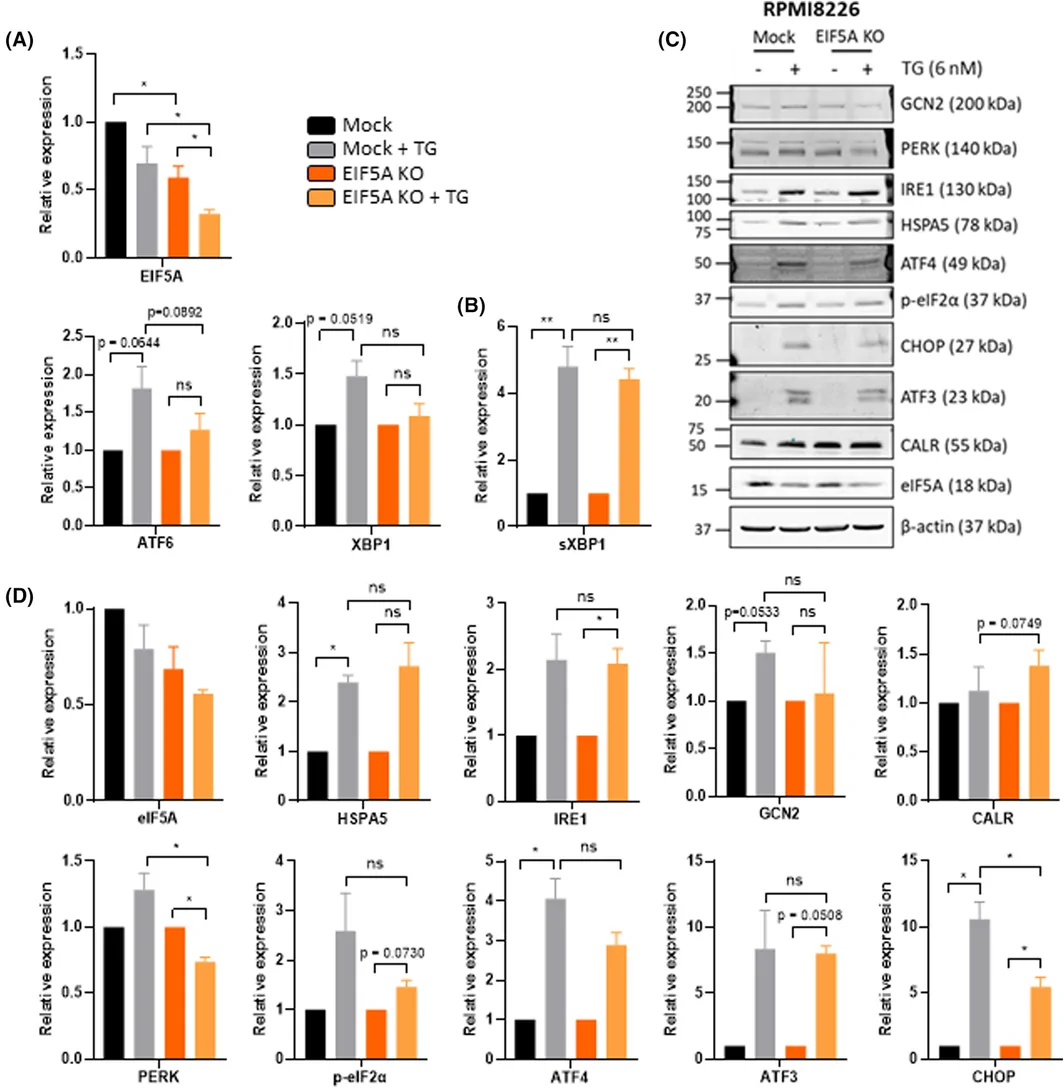
Unfolded protein response (UPR) protein expression in mock vs *EIF5A* knockout (KO) cells upon thapsigargin treatment. (A) Gene expression of key UPR proteins in RPMI‐8226 mock and *EIF5A* KO cells after TG treatment (24 h, 6 nm) determined by RNA sequencing, *N* = 4. Data are presented as mean ± SEM. *P*‐values were calculated using paired *t*‐tests, *P < 0.05. (B) XBP1 splicing in RPMI‐8226 mock and *EIF5A* KO cells after TG treatment (24 h, 6 nm) determined by qPCR, *N* = 4. Data are presented as mean ± SEM. *P*‐values were calculated using paired *t*‐tests, **P < 0.01. (C, D) Western blot (C) and quantification (D) of key UPR proteins in RPMI‐8226 mock and *EIF5A* KO cells after TG treatment (24 h, 6 nm) *N* = 3. Data are presented as mean ± SEM. *P*‐values were calculated using paired *t*‐tests, *P < 0.05. TG, thapsigargin.

### 3.5 Inhibition of eIF5A hypusination recapitulates thapsigargin resistance

eIF5A is uniquely activated by the post‐translational modification hypusination, which is a two‐step process carried out by enzymes DHPS and DOHH [21, 24, 35]. Hypusination can be pharmacologically inhibited by GC7, a competitive and reversible inhibitor of DHPS [23]. In RPMI‐8226 cells, treatment with GC7 reduced hypusination by half after 24 h, but levels were restored at 48 h, whereas eIF5A hypusination continued to decrease between 24 and 48 h in LP‐1 cells (Fig. 5A,B, Fig. S5). To investigate the effect of eIF5A inhibition on basal UPR activation, we measured UPR induction upon GC7 treatment (Fig. 5C). Consistent with the temporal hypusination response, RPMI‐8226 cells displayed minimal UPR activation upon GC7 treatment, with a modest increase of CHOP protein levels at 24 h of GC7 treatment. In contrast, LP‐1 cells showed a strong upregulation of CHOP at both 24 and 48 h and a slight increase in ATF4 at 24 h. This response occurred independently of eIF2α, as no increase in phosphorylation was detected. To determine whether the protection to thapsigargin‐induced cell death observed in *EIF5A* KO cells was dependent on eIF5A hypusination, RPMI‐8226 and LP‐1 cells were co‐treated with GC7 for 24 or 48 h. In RPMI‐8226 cells, GC7 pretreatment failed to enhance survival to thapsigargin (Fig. 5D). In LP‐1 cells, the addition of GC7 did reduce thapsigargin‐induced cell death both in the absence and presence of caspase inhibition. To check whether eIF5A activity was differentially affected between RPMI‐8226 and LP‐1 cell lines in the presence of thapsigargin, we investigated whether thapsigargin treatment directly affected eIF5A hypusination. Thapsigargin specifically reduced eIF5A hypusination in RPMI‐8226 cells, whereas bortezomib did not have an effect (Fig. 5F,G). Notably, in LP‐1 cells, eIF5A hypusination was unaffected by thapsigargin (Fig. 5H). Together, these data indicate that GC7 and thapsigargin may both affect eIF5A hypusination, which may contribute to the level of UPR activation and thapsigargin‐induced cell death, highlighting cell‐line‐specific effects on eIF5A activity and subsequent cellular outcome.

**Fig. 5:**
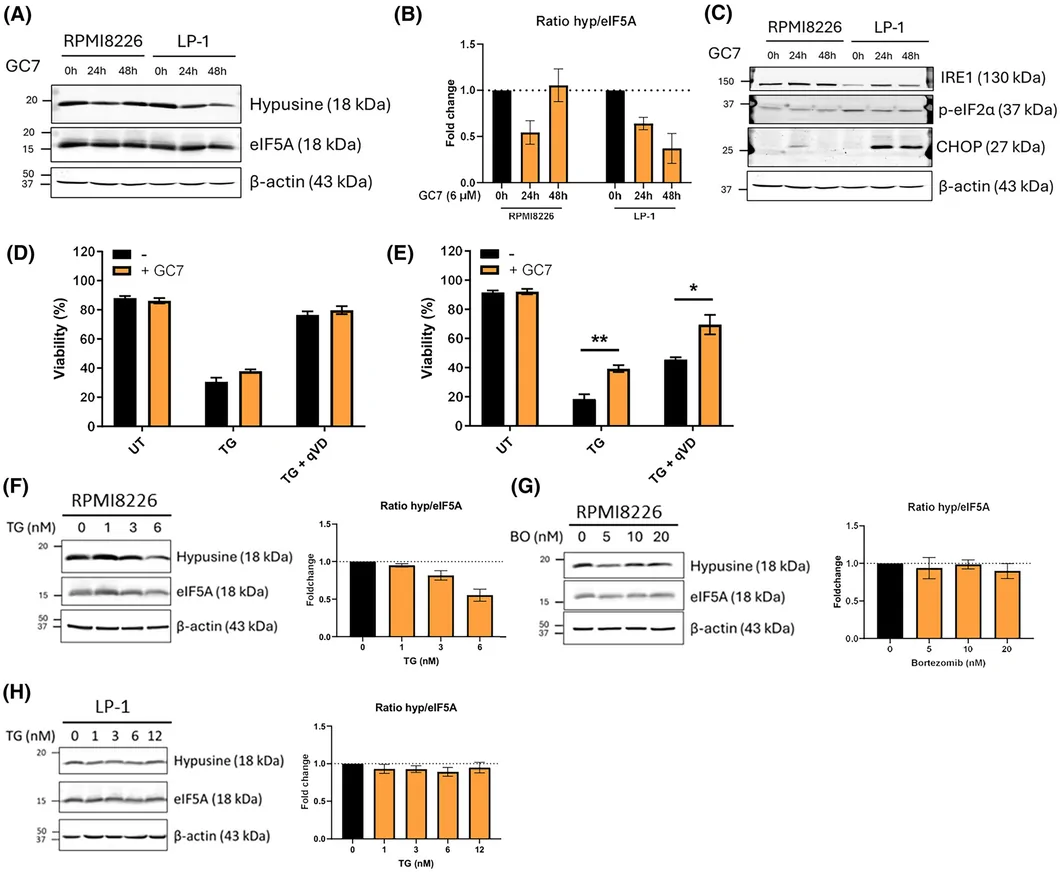
Inhibiting eIF5A hypusination and unfolded protein response (UPR) activity. (A) Western blot of eIF5A hypusination in RPMI‐8226 and LP‐1 cells treated with GC7 (6 μm), *N* = 3, one representative experiment is shown. (B) Quantification of A. Data are presented as mean ± SEM. (C) Expression of key UPR proteins upon in RPMI‐8226 and LP‐1 cells treated with GC7 (6 μm), *N* = 2, one representative experiment is shown. (D) Viability of RPMI‐8226 cells treated with TG (6 nm), GC7 (6 μm), and qVD (5 μm) for 24 h. Data are presented as mean ± SEM, *N* = 3. (E) Viability of LP‐1 cells treated with TG (6 nm), GC7 (6 μm), and qVD (5 μm) for 48 h. Data are presented as mean ± SEM, *N* = 3. (F) Western blot of eIF5A hypusination in RPMI8226 mock cells after TG treatment (24 h, 6 nm), *N* = 4, one representative experiment is shown. Data are presented as mean ± SEM. (G) Western blot of eIF5A hypusination in RPMI8226 cells treated with BO (24 h, 20 nm), *N* = 3, one representative experiment is shown. Data are presented as mean ± SEM. (H) Western blot of eIF5A hypusination in LP‐1 mock cells after TG treatment (24 h, 12 nm), *N* = 4, one representative experiment is shown. Data are presented as mean ± SEM. *P*‐values were calculated using student's *t*‐tests, *P < 0.05, **P < 0.01. TG, thapsigargin.

### 3.6 Feedback regulation of thapsigargin on eIF5A inhibition

To further explore how thapsigargin may directly affect eIF5A hypusination in RPMI‐8226 cells, we investigated the expression of the two enzymes responsible for eIF5A hypusination, DHPS and DOHH [21]. Thapsigargin treatment significantly reduced DOHH protein levels, whereas DHPS expression was unaffected (Fig. 6A). To explore whether reduced DHPS and DOHH enzyme expression could protect cells to thapsigargin‐induced cell death, CRISPR‐Cas9 KOs were generated (Fig. 6B). Functionally, depletion of either DHPS and DOHH conferred protection against thapsigargin‐induced cell death, indicating that modulation of eIF5A hypusination may directly affect thapsigargin sensitivity (Fig. 6C). Thapsigargin treatment did not reduce transcription of DHPS and DOHH (Fig. 6D) in mock cells, though it did lower transcription in *EIF5A* KO cells. Together, these findings are compatible with a model in which thapsigargin induces UPR activation that can promote cell death, while also affecting eIF5A activity that leads to dampen UPR signaling and subsequent cell death (Fig. 6E). Overall, our findings suggest a previously unrecognized feedback mechanism in which thapsigargin‐mediated modulation of eIF5A helps shape ER stress responses and cell fate.

**Fig. 6:**
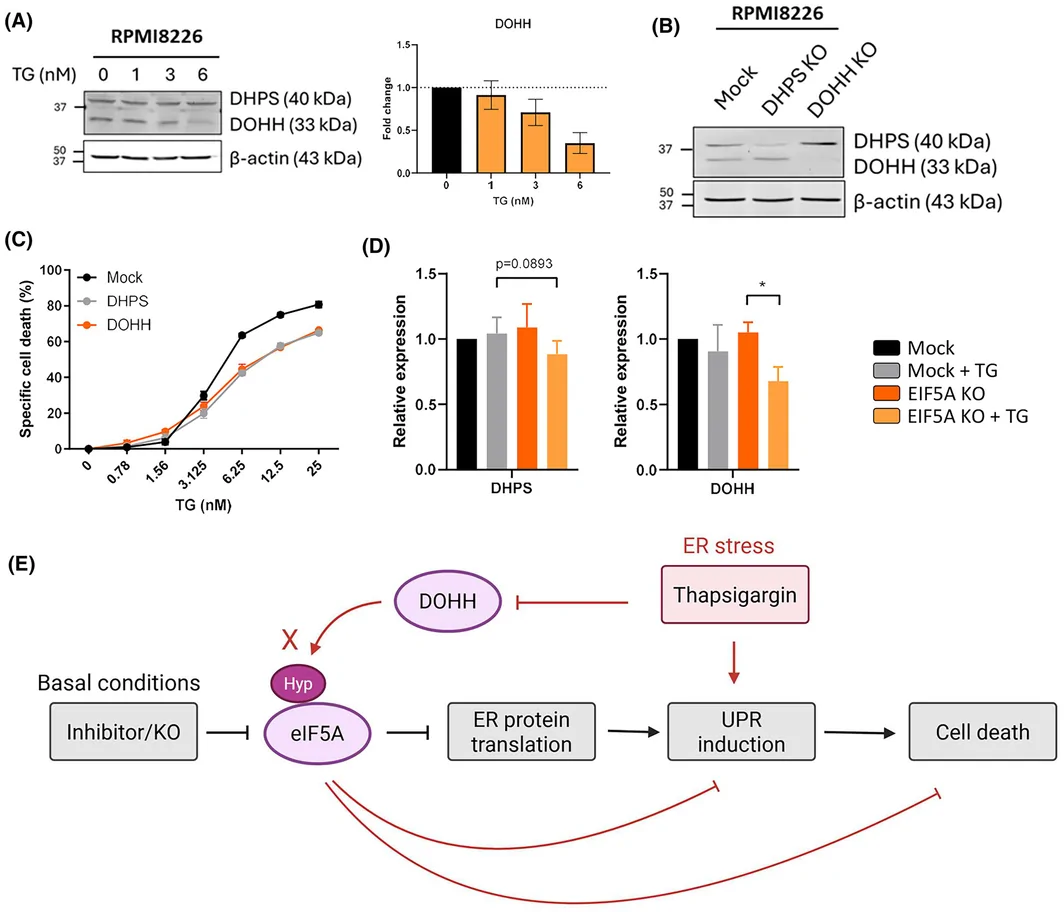
Feedback mechanism between thapsigargin and eIF5A hypusination. (A) Western blot and quantification of DOHH and DHPS in RPMI‐8226 mock cells after TG treatment (24 h, 6 nm), *N* = 3, one representative experiment is shown. Data are presented as mean ± SEM. (B) Western Blot of DHPS and DOHH knockout (KO) verification in RPMI‐8226 cells. (C) Specific cell death of RPMI‐8226 mock and DHPS and DOHH KO cells treated with TG for 24 h, *N* = 3. (D) Gene expression of DHPS and DOHH in RPMI‐8226 mock and *EIF5A* KO cells after TG treatment (24 h, 6 nm) determined by RNA sequencing, *N* = 4. Data are presented as mean ± SEM. *P*‐values were calculated using paired *t*‐tests, *P < 0.05, **P < 0.01; ***P < 0.001. (E) Proposed model of feedback mechanism between thapisgargin treatment and eIF5A hypusination. Under basal conditions, eIF5A depletion may reduce ER‐associated protein translation, leading to ER stress, unfolded protein response (UPR) activation and cell death. During prolonged thapsigargin‐induced ER stress, loss of eIF5A confers a proliferative advantage and reduces cell death. Thapsigargin may inhibit eIF5A hypusination via DOHH, suggesting a feedback mechanism. TG, thapsigargin.

## 4 Discussion

In conclusion, our results demonstrate a novel modulatory role for eIF5A in the thapsigargin‐mediated ER stress response in MM cell lines by altering translational regulation of UPR transcripts. eIF5A is a highly conserved and abundant protein and fulfills diverse roles in the cell, for instance in metabolism, protein translation, stress granules and the cytoskeleton [21, 24, 36, 37]. Loss of eIF5A expression impairs mouse embryonic development [38] and is lethal in many cell types [32]. Consistent with KO efficiency, we were not able to reduce eIF5A protein and hypusination levels for more than half without inducing lethality. This incomplete suppression likely limited our ability to fully resolve the contribution of eIF5A as well as its hypusination to the ER stress response. The growth disadvantage of *EIF5A* KO cells was not caspase‐dependent and thus points toward an effect on proliferation, which is supported by other studies describing decreased proliferation in the absence of eIF5A [39, 40, 41, 42]. For instance, GC7 treatment for longer periods was found to inhibit cell proliferation, likely due to eIF5A being required for translation of transcripts involved in cell cycle progression, specifically from G1‐to‐S phase [41, 42, 43].

Earlier studies show that under basal conditions eIF5A depletion or hypusination inhibition induces UPR signaling [21, 44], which we also observed in LP‐1 cells. This basal UPR induction could be explained by the accumulation of unfolded proteins in the ER resulting from impaired translation. This could also explain why B cells are more sensitive to eIF5A depletion compared with other cell types [32], since they depend on proper UPR function for the secretion of immunoglobulins.

In contrast to the role of eIF5A under basal conditions, loss of eIF5A resulted in a proliferation benefit compared with WT cells upon prolonged ER stress induced by thapsigargin. Upon short‐term thapsigargin treatment, eIF5A depletion also reduced treatment‐induced cell death. These findings might be explained by differences in translation specifically in the context of thapsigargin treatment. Thapsigargin reduced general translation in mock cells, but did not significantly reduce translation in *EIF5A* KO cells, suggesting an altered translational stress response in KO and mock cells. ER stress inhibits general translation, but allows the translation of stress‐responsive transcripts [7]. Translation by ribosomes on the ER lumen continues upon ER stress, likely to synthesize proteins necessary for cell repair [45]. eIF5A co‐localizes with ER‐associated proteins under stress conditions, supporting a role for eIF5A in the translation of ER stress‐related mRNAs [45, 46]. Consistent with our data, a study performed in pancreatic β cells showed that GC7 treatment reduced the protein folding capacity of the ER and decreased CHOP synthesis upon thapsigargin treatment, which was directly linked to reduced thapsigargin‐induced cell death [46].

In general, eIF5A is described to be an elongation factor, supporting the translation of transcripts containing specific motifs, such as polyproline stretches, and does not play a major role in general translation [37, 41, 47]. This fits with our data, as we did not observe a significant reduction of translation levels upon loss of eIF5A, but rather a shift in translational regulation of specific transcripts. Loss of eIF5A was accompanied by the loss of translation of many transcripts involved in translation and ER stress, corresponding with its role in the synthesis of ER proteins [45, 46, 48]. Consistent with these findings, eIF5A‐deficient cells displayed an attenuated thapsigargin‐mediated UPR induction compared with mock cells. Given the dual role of the UPR in promoting both cell survival and cell death, its inhibition may contribute to increased survival of eIF5A‐deficient cells under ER stress conditions. A small fraction of transcripts was uniquely translated in *EIF5A* KO cells upon thapsigargin treatment, and included the chaperone calreticulin, suggesting a specific regulation mechanism. Consistently, calreticulin was previously found to have a significantly increased protein expression upon eIF5A depletion [44]. In turn, cells with higher calreticulin expression have a higher buffering capacity of ER‐lumenal calcium [49], which may dampen the amplitude of thapsigargin‐induced calcium depletion. This may decrease the protein misfolding burden and thus reduce activation of the UPR response. Since calreticulin buffers a significant proportion of calcium stored in the ER lumen [49], it is plausible that translational upregulation of calreticulin upon eIF5A depletion may specifically protect cells to thapsigargin and not to other ER stress inducers.

Besides attenuated translation of specific transcripts, eIF5A depletion affected the translational regulation of transcripts within the polysomal fraction. In mock cells treated with thapsigargin, many polysome‐associated transcripts were found in the translation category, decoupling translation from transcription and thereby allowing for rapid changes in protein levels, a common characteristic of stress response genes [50]. In *EIF5A* KO cells, this fraction was considerably smaller and a larger group of transcripts was found in the buffering category, indicating that protein levels of these transcripts are stabilized regardless of changes in transcription. Consistent with the role of eIF5A in promoting translation of polyproline motifs [33], we confirmed enrichment of polyproline stretches in transcripts in the translation category for mock cells, yet in the buffering category for KO cells. These shifts in translational regulation suggest that eIF5A‐depleted cells may have a less rapid adjustment in the synthesis of UPR proteins upon thapsigargin, consistent with reduced protein levels. Additionally, KO cells showed a more buffered translational response to acute ER stress, potentially prioritizing translation of survival genes as a coping mechanism. Moreover, some transcripts, including calreticulin, were only translated in *EIF5A* KO cells, suggesting a specific stress response in these cells. Aforementioned polyproline‐rich transcripts did not indicate a clear association with the UPR. However, many of these transcripts shared a functional pattern in nuclear gene regulation and RNA processing, suggesting potential indirect links to ER stress that remain to be clarified. Further research will be required to determine how eIF5A‐dependent translational regulation contributes to ER stress‐related pathways.

The general consensus is that eIF5A activity requires the post‐translational modification hypusination [21, 22, 35]. Based on collective CRISPR screen data, one of the top co‐dependencies of eIF5A is DOHH [32], which is also supported by our data and underlies the importance of active eIF5A hypusination. Notably, inhibition of hypusination by GC7 did not protect RPMI‐8226 cells to thapsigargin‐induced cell death as observed in *EIF5A* KO cells. A potential confounder could be that thapsigargin treatment by itself inhibits eIF5A hypusination in RPMI‐8226 cells, which may hinder additional inhibition by GC7 and therefore not result in any GC7‐mediated protection in the presence of thapsigargin. This is corroborated by the observation that thapsigargin‐induced cell death in LP‐1 cells was reduced by addition of GC7, in which thapsigargin did not directly affect eIF5A hypusination. This discrepancy could be attributed to the fact that RPMI‐8226 and LP‐1 cells exhibit cell‐line‐specific differences in ER stress responses and subsequent compensation mechanisms. Furthermore, we and others showed that timing and dynamics play a role in the efficacy of GC7 [51], which may also be dependent on cellular spermidine levels, as spermidine serves as the essential substrate in the enzymatic hypusination process by DHPS and DOHH [51]. Yet, we verified that spermidine levels are not significantly different between both cell lines (Fig. S6). Altogether, directly comparing data from models with different dependencies and sensitivities might not be valid in this context.

The importance of eIF5A hypusination in this model is indicated by the reduction of hypusination levels upon thapsigargin treatment, suggesting a feedback mechanism, in which thapsigargin treatment reduced expression of DOHH, and KO of either DHPS or DOHH resulted in better survival under thapsigargin treatment. By inhibiting eIF5A hypusination, thapsigargin could lower UPR induction and cell death, thereby diminishing the ensuing UPR response. This feedback mechanism could provide a window for fine‐tuning the balance between cell survival and cell death. The study of Robbins et al. [46] previously described a feedback mechanism in pancreatic β cells, where thapsigargin enhanced the hypusination and activity of DHPS. Although in a different direction, this indicates interactions between thapsigargin‐induced ER stress and eIF5A regulation, which could be cell type‐dependent. Moreover, *EIF5A* KO affected UPR mediators at both protein and transcriptional levels, suggesting indirect effects as well. The effects of eIF5A depletion were specific for thapsigargin, as we and others described that loss of eIF5A sensitizes to bortezomib treatment in MM cells [52]. Consistent with these data, only thapsigargin reduced eIF5A hypusination. A possible explanation could be that thapsigargin and bortezomib induce divergent UPR responses, and eIF5A activity might be specifically involved in calcium‐dependent ER stress.

## 5 Conclusion

In conclusion, our study aimed to dissect the molecular mechanisms that determine whether ER stress triggers repair or cell death in MM cells. We found that loss of eIF5A protects MM cells to thapsigargin‐induced cell death, in which eIF5A contributes to the translational regulation of specific transcripts, including those involved in the thapsigargin‐mediated UPR stress response. These findings provide new insights into the translational control of ER stress responses in MM cells and identify eIF5A as a potential node for therapeutical intervention. Modulating eIF5A activity, alone or in combination with ER stress‐inducing agents, may be a strategy to overcome drug resistance to current therapies.

## Conflict of interest

The authors declare no conflict of interest.

## Author contributions

DB contributed to the experiments, data analysis, and manuscript drafting. ID contributed to the experiments and data analyses. RV, NEH, ML, and PC assisted with polysome profiling, data analysis, and manuscript editing. EE contributed to the conceptualization, data analyses, and writing. MH contributed to the data analyses, concepts, and manuscript revision.

## Supporting information


**Fig. S1.** EIF5A gRNA validation.

**Fig. S2.** CRISPR Screen JeKo‐1 cells.

**Fig. S3.** Gene expression of key UPR proteins.

**Fig. S4.** Expression of apoptotic proteins in mock vs EIF5A KO cells upon thapsigargin treatment.

**Fig. S5.** Hypusination and eIF5A western blot quantification.

**Fig. S6.** Spermidine ELISA.

**Table S1.** Targeted CRISPR‐Cas9 screen.

**Table S2.** sgRNA sequences.

**Table S3.** Antibodies.

**Table S4.** qPCR primers.

## Data Availability

All data included in the manuscript is available on request from the corresponding author.
