# Supplementary material for: A role for eIF5A in translational control of stress‐inducible proteins in multiple myeloma cell lines
Source: Mol Oncol. 2026 Oct 21:10.1002/1878-0261.70328. Online ahead of print. doi: 10.1002/1878-0261.70328 (PMC13685402; doi:10.1002/1878-0261.70328)
Supplement: Supplementary file 1 [file MOL2-9999-0-s002.docx]

Supplementary Table 4: qPCR primers

| **Gene name** | **Forward primer** | **Reverse primer** |
| --- | --- | --- |
| *Spliced XBP1* | TGCTGAGTCCGCAGCAGG | GTCCAGAATGCCCAACAGGA |
