# Supplementary material for: A role for eIF5A in translational control of stress‐inducible proteins in multiple myeloma cell lines
Source: Mol Oncol. 2026 Oct 21:10.1002/1878-0261.70328. Online ahead of print. doi: 10.1002/1878-0261.70328 (PMC13685402; doi:10.1002/1878-0261.70328)
Supplement: Supplementary file 2 [file MOL2-9999-0-s003.docx]

Supplementary Table 3: antibodies

| **Target** | **Supplier** | **Cat number** |
| --- | --- | --- |
| eIF5A | BD | 611977 |
| β-actin | Biolegend | 664801 |
| GCN2 | Cell Signaling | 65981 |
| PERK | Cell Signaling | 5683 |
| IRE1α | Cell Signaling | 3294 |
| HSPA5 | Cell Signaling | 3177 |
| ATF4 | Cell Signaling | 11815 |
| p-eIF2α | Cell Signaling | 3398 |
| CHOP | Cell Signaling | 2895 |
| ATF3 | Cell Signaling | 33593 |
| MCL1 | Abcam | ab32087 |
| BCL2 | Cell Signaling | 15071 |
| BAX | Cell Signaling | 5023 |
| BCL-XL | Cell Signaling | 2764 |
| BIM | StressMarq | SPC-113D |
| NOXA | Novus Biologicals | NB600-1159 |
| Hypusine | ProteoGenix | PTX18841 |
| DHPS | Santa Cruz | sc-365077 |
| DOHH | Santa Cruz | sc-271868 |
| IRDye 680LT Donkey anti-Mouse IgG Secondary Antibody | Li-Cor | 926-68022 |
| IRDye 800CW Donkey anti-Mouse IgG Secondary Antibody | Li-Cor | 926-32212 |
| IRDye 680LT Donkey anti-Rabbit IgG Secondary Antibody | Li-Cor | 926-68023 |
| IRDye 800CW Donkey anti-Rabbit IgG Secondary Antibody | Li-Cor | 926-32213 |
| IRDye 680 Goat anti-Rat IgG Secondary Antibody | Li-Cor | 926-32229 |
| IRDye 800CW Goat anti-Rat IgG Secondary Antibody | Li-Cor | 926-32219 |
