# Supplementary material for: A role for eIF5A in translational control of stress‐inducible proteins in multiple myeloma cell lines
Source: Mol Oncol. 2026 Oct 21:10.1002/1878-0261.70328. Online ahead of print. doi: 10.1002/1878-0261.70328 (PMC13685402; doi:10.1002/1878-0261.70328)
Supplement: Supplementary file 3 [file MOL2-9999-0-s005.docx]

Supplementary Table 2: sgRNA sequences

| **Gene name** | **Target sequence** | **Exon** |
| --- | --- | --- |
| AAV1S locus (Mock) | CCTCTAAGGTTTGCTTACGA | n.a. |
| EIF5A sgRNA 1 | GTGAAATTCTAACCTTGGCG | 2 |
| EIF5A sgRNA 2 | TCTCGACGATCTTACATGGC | 2 |
| EIF5A sgRNA 3 | TACATACAGGTCCATCTGGT | 3 |
| DHPS sgRNA | GATGGAGCAGAACACAGAGG | 4 |
| DOHH sgRNA | GTCCACCAGCGTCTGCCCGA | 2 |
