# Supplementary material for: A role for eIF5A in translational control of stress‐inducible proteins in multiple myeloma cell lines
Source: Mol Oncol. 2026 Oct 21:10.1002/1878-0261.70328. Online ahead of print. doi: 10.1002/1878-0261.70328 (PMC13685402; doi:10.1002/1878-0261.70328)
Supplement: Supplementary file 4 [file MOL2-9999-0-s007.docx]

Supplementary Table 1: Targeted CRISPR-Cas9 screen

| **Protein** | **Gene** | **Category** |
| --- | --- | --- |
| BIP/GRP78 | HSPA5 | Classical UPR |
| Grp170 | HYOU1 | Classical UPR |
| PERK | EIF2AK3 | Classical UPR |
| eIF2a | EIF2A | Classical UPR |
| ATF4 | ATF4 | Classical UPR |
| CHOP | DDIT3 | Classical UPR |
| GADD34 | PPP1R15A | Classical UPR |
| PP1 | PP1CA | Classical UPR |
| IRE1 | ERN1 | Classical UPR |
| XBP1 | XBP1 | Classical UPR |
| RTCB | RTCB | Classical UPR |
| JNK1 | MAPK8 | Classical UPR |
| TRAF2 | TRAF2 | Classical UPR |
| p38 mapk | MAPK14 | Classical UPR |
| ASK1 | MAP3K5 | Classical UPR |
| JNK2 | MAPK9 | Classical UPR |
| ATF6 | ATF6 | Classical UPR |
| S1P | S1PR1 | Classical UPR |
| S2P | MBTPS2 | Classical UPR |
| OASIS | CREB3L1 | Classical UPR |
| CREB | CREB1 | Classical UPR |
| CREB-H | CREB3L3 | Classical UPR |
| Luman | CREB3 | Classical UPR |
| BBF2H7 | CREB3L2 | Classical UPR |
| ATF3 | ATF3 | Classical UPR |
| ERK2/MAPK1 | MAPK1 | Classical UPR |
| Casp 2 | CASP2 | UPR cell death |
| Casp 8 | CASP8 | UPR cell death |
| Casp 10 | CASP10 | UPR cell death |
| Casp 12 | CASP12 | UPR cell death |
| DR5 | TNFRSF10B | UPR cell death |
| DR4 | TNFRSF10A | UPR cell death |
| CD95/Fas | FAS | UPR cell death |
| TNFR1 | TNFRSF1A | UPR cell death |
| cFLIP | CFLAR | UPR cell death |
| TRADD | TRADD | UPR cell death |
| CD95L/FasL | FASLG | UPR cell death |
| TRAIL | TNFSF10 | UPR cell death |
| TNF | TNF | UPR cell death |
| RNF183 | RNF183 | UPR cell death |
| XIAP | XIAP | UPR cell death |
| NRF2 | NFE2L2 | Redox |
| KEAP1 | KEAP1 | Redox |
| HO-1 | HMOX1 | Redox |
| P62 | SQSTM1 | Redox |
| ERO1α | ERO1A | Redox balance |
| PDIA3/Erp57 | PDIA3 | Redox balance |
| GSH | GSS | Redox balance |
| TXN | TXN | Redox balance |
| NADPH oxidase 4 | NOX4 | Redox balance |
| NADPH-P450 reductase | POR | Redox balance |
| ERO1β | ERO1B | Redox balance |
| VCP/p97 | VCP | ERAD |
| NGLY1 | NGLY1 | ERAD |
| UGGT | UGGT1 | ERAD |
| ERMan1 | MAN1B1 | ERAD |
| Man1C1 | MAN1C1 | ERAD |
| OS9 | OS9 | ERAD |
| XTP3-B/Erlectin | ERLEC1 | ERAD |
| LONP2 | LONP2 | ERAD |
| CPVL | CPVL | ERAD |
| SEL1L | SEL1L | ERAD |
| Hrd1 | SYVN1 | ERAD |
| ERFAD | FOXRED2 | ERAD |
| Erp90 | TXNDC16 | ERAD |
| Derlin-1 | DERL1 | ERAD |
| Derlin-2 | DERL2 | ERAD |
| AUP1 | AUP1 | ERAD |
| UBXD8 | FAF2 | ERAD |
| VIMP | SELENOS | ERAD |
| Herp | HERPUD1 | ERAD |
| Erlin-1 | ERLIN1 | ERAD |
| Erlin-2 | ERLIN2 | ERAD |
| TMUB1 | TMUB1 | ERAD |
| Gp78 | AMFR | ERAD |
| Ufd1 | UFD1 | ERAD |
| NPL4 | NPLOC4 | ERAD |
| Fbx2 | FBXO2 | ERAD |
| Fbx6 | FBXO6 | ERAD |
| Skp1 | SKP1 | ERAD |
| Skp2 | SKP2 | ERAD |
| Cullin | CUL1 | ERAD |
| FBXO46 | FBXO46 | ERAD |
| Roc-1 | RBX1 | ERAD |
| iRhom1 | RHBDF1 | ERAD |
| iRhom2 | RHBDF2 | ERAD |
| UBAC1 | UBAC1 | ERAD |
| UBXD8 | FAF2 | ERAD |
| Ufd1 | UFD1 | ERAD |
| Npl4 | NPLOC4 | ERAD |
| Yod1 | YOD1 | ERAD |
| VCIP135 | VCPIP1 | ERAD |
| USP19 | USP19 | ERAD |
| Ataxin-3 | ATXN3 | ERAD |
| Ube4a | UBE4A | ERAD |
| EDEM | EDEM1 | ERAD |
| EDEM2 | EDEM2 | ERAD |
| EDEM3 | EDEM3 | ERAD |
| BRI3BP | BRI3BP | ERAD |
| HM13/SPP | HM13 | ERAD |
| UBE2G2 | UBE2G2 | ERAD |
| TMEM201 | TMEM201 | ERAD |
| TMEM43 | TMEM43 | ERAD |
| LRRC59 | LRRC59 | ERAD |
| CLPTM1 | CLPTM1 | ERAD |
| Ubac2 | UBAC2 | ERAD |
| MDM1 | MDM1 | Autophagy |
| SNX13 | SNX13 | Autophagy |
| CaMKK | CAMKK2 | Autophagy |
| mTor | MTOR | Autophagy |
| ULK1 | ULK1 | Autophagy |
| Atg101 | ATG101 | Autophagy |
| Atg13 | ATG13 | Autophagy |
| FIP200 | RB1CC1 | Autophagy |
| UVRAG | UVRAG | Autophagy |
| Vps34 | PIK3C3 | Autophagy |
| Ambra1 | AMBRA1 | Autophagy |
| Atg5 | ATG5 | Autophagy |
| Atg12 | ATG12 | Autophagy |
| Atg3 | ATG3 | Autophagy |
| Atg7 | ATG7 | Autophagy |
| LC3A | MAP1LC3A | Autophagy |
| LC3B | MAP1LC3B | Autophagy |
| BLOS1 | BLOC1S1 | Autophagy |
| Atg16L1 | ATG16L1 | Autophagy |
| NBR1 | NBR1 | Autophagy |
| ALFγ | GTF2A1L | Autophagy |
| Atg1 | ATG4A | Autophagy |
| Atg2 | ATG4B | Autophagy |
| Atg3 | ATG4C | Autophagy |
| Atg4 | ATG4D | Autophagy |
| Atg8a | GABARAP | Autophagy |
| AMPK | PRKAA2 | Autophagy |
| PKC | PRKCA | Autophagy |
| Bcl-2 | BCL2 | Bcl-2 family |
| Bcl-Xl | BCL2L1 | Bcl-2 family |
| Bcl-W | BCL2L2 | Bcl-2 family |
| Mcl-1 | MCL1 | Bcl-2 family |
| Bfl-1 | BCL2A1 | Bcl-2 family |
| Bcl-B | BCL2L10 | Bcl-2 family |
| Noxa | PMAIP1 | Bcl-2 family |
| Puma | BBC3 | Bcl-2 family |
| Bim | BCL2L11 | Bcl-2 family |
| Bid | BID | Bcl-2 family |
| Bax | BAX | Bcl-2 family |
| Bak | BAK1 | Bcl-2 family |
| Bad | BAD | Bcl-2 family |
| Bmf | BMF | Bcl-2 family |
| BNIP-1 | BNIP1 | Bcl-2 family |
| BNIP-3 | BNIP3 | Bcl-2 family |
| AP31 | AP31 | Bcl-2 family |
| Bcl-G | BCL2L13 | Bcl-2 family |
| BCL-RAMBO | BCL2L13 | Bcl-2 family |
| Bok | BOK | Bcl-2 family |
| Spike | SPIKE | Bcl-2 family |
| HRK | HRK | Bcl-2 family |
| Bik | BIK | Bcl-2 family |
| Beclin-1 | BECN1 | Bcl-2 family |
| BFK | BCL2L15 | Bcl-2 family |
| Bap31 | BCAP31 | Bcl-2 family |
| NIX | BNIP3L | Bcl-2 family |
| LAMP2 | LAMP2 | Ferroptosis |
| GPX4 | GPX4 | Ferroptosis |
| Squalene synthetase | FDFT1 | Ferroptosis |
| SLC7A11 | SLC7A11 | Ferroptosis |
| SLC3A2 | SLC3A2 | Ferroptosis |
| VDAC1 | VDAC1 | Ferroptosis |
| VDAC2 | VDAC2 | Ferroptosis |
| VDAC3 | VDAC3 | Ferroptosis |
| P53 | TP53 | Ferroptosis |
| SAT1 | SAT1 | Ferroptosis |
| CDKN1A/P21 | CDKN1A | Ferroptosis |
| ALOX15 | ALOX15 | Ferroptosis |
| AIFM-1 | AIFM1 | Ferroptosis |
| FSP/AIFM2 | AIFM2 | Ferroptosis |
| TFRC (TFR1) | TFRC | Ferroptosis |
| HSPB1 | HSPB1 | Ferroptosis |
| FTL | FTL | Ferroptosis |
| FTH1 | FTH1 | Ferroptosis |
| Ceruloplasmin | CP | Ferroptosis |
| Transferrin | TF | Ferroptosis |
| STEAP3 | STEAP3 | Ferroptosis |
| DMT1 | SLC11A2 | Ferroptosis |
| ZIP8/14 | SLC39A8 | Ferroptosis |
| FPN1 | SLC40A1 | Ferroptosis |
| DPP4 | DPP4 | Ferroptosis |
| ACSL4 | ACSL4 | Ferroptosis |
| LPCAT3 | LPCAT3 | Ferroptosis |
| LOX | LOX | Ferroptosis |
| NCOA4 | NCOA4 | Ferroptosis |
| BAP1 | BAP1 | Ferroptosis |
| NOX2 | CYBB | Ferroptosis |
| ABCA1 | ABCA1 | Ferroptosis |
| SREBP2 | SREBF2 | Ferroptosis |
| HMG-CoA reductase | HMGCR | Ferroptosis |
| HIF2a | EPAS1 | Ferroptosis |
| HELLS | HELLS | Ferroptosis |
| PHD2/EGLN1 | EGLN1 | Ferroptosis |
| HIF1A | HIF1A | Ferroptosis |
| ZEB1 | ZEB1 | Ferroptosis |
| IL-6 | IL6 | Ferroptosis |
| IL-1B | IL1B | Ferroptosis |
| iNOS | NOS2 | Ferroptosis |
| LTB4-R1 | LTB4R | Ferroptosis |
| HMGB1 | HMGB1 | Ferroptosis |
| RAGE | AGER | Ferroptosis |
| SCP2 | SCP2 | Ferroptosis |
| AKR1C1 | AKR1C1 | Ferroptosis |
| AKR1C2 | AKR1C2 | Ferroptosis |
| AKR1C3 | AKR1C3 | Ferroptosis |
| AKR1C4 | AKR1C4 | Ferroptosis |
| ACC1 | ACACA | Ferroptosis |
| CARS | CARS1 | Ferroptosis |
| ATP5G3 | ATP5MC3 | Ferroptosis |
| EMC2/TTC35 | EMC2 | Ferroptosis |
| PTGES2 | PTGES2 | Ferroptosis |
| CHAC1 | CHAC1 | Ferroptosis |
| CDO1 | CDO1 | Ferroptosis |
| NFS1 | NFS1 | Ferroptosis |
| MUC-1C | MUC1 | Ferroptosis |
| SLC1A5 | SLC1A5 | Ferroptosis |
| MT-1G | MT1G | Ferroptosis |
| CISD1 | CISD1 | Ferroptosis |
| Glutamate-Ammonia Ligase | GLUL | Ferroptosis |
| CISD2 | CISD2 | Ferroptosis |
| RPL8 | RPL8 | Ferroptosis |
| IREB2 | IREB2 | Ferroptosis |
| CS | CS | Ferroptosis |
| ACSF2 | ACSF2 | Ferroptosis |
| NLRP3 | NLRP3 | Pyroptosis |
| NEK7 | NEK7 | Pyroptosis |
| NAIP | NAIP | Pyroptosis |
| Casp 1 | CASP1 | Pyroptosis |
| GSDMA | GSDMA | Pyroptosis |
| GSDMB | GSDMB | Pyroptosis |
| GSDMC | GSDMC | Pyroptosis |
| GSDMD | GSDMD | Pyroptosis |
| GSDME | GSDME | Pyroptosis |
| Casp 4 | CASP4 | Pyroptosis |
| Casp 5 | CASP5 | Pyroptosis |
| NLRP1 | NLRP1 | Pyroptosis |
| NLRC2 | NLRC2 | Pyroptosis |
| ASC | PYCARD | Pyroptosis |
| IL-1B | IL1B | Pyroptosis |
| IL-18 | IL18 | Pyroptosis |
| Pyrin | MEFV | Pyroptosis |
| AIM2 | AIM2 | Pyroptosis |
| TRAF2 | TRAF2 | Necroptosis |
| TRAF5 | TRAF5 | Necroptosis |
| RIPK1 | RIPK1 | Necroptosis |
| cIAP1 | BIRC2 | Necroptosis |
| cIAP2 | BIRC3 | Necroptosis |
| LUBAC | RNF31 | Necroptosis |
| CYLD | CYLD | Necroptosis |
| TRAF3 | TRAF3 | Necroptosis |
| TRAF6 | TRAF6 | Necroptosis |
| FADD | FADD | Necroptosis |
| RIPK3 | RIPK3 | Necroptosis |
| MLKL | MLKL | Necroptosis |
| A20/TNFAIP3 | TNFAIP3 | Necroptosis |
| TAK1 | MAP3K7 | Necroptosis |
| TAB2 | TAB2 | Necroptosis |
| TAB3 | TAB3 | Necroptosis |
| RFK | RFK | Necroptosis |
| p22phox | CYBA | Necroptosis |
| NOX1 | NOX1 | Necroptosis |
| Cezanne | OTUD7B | Necroptosis |
| USP21 | USP21 | Necroptosis |
| PGAM5 L/S | PGAM5 | Necroptosis |
| Drp1 | DNM1L | Necroptosis |
| IKK1 | CHUK | Necroptosis |
| IKK2 | IKBKB | Necroptosis |
| NEMO | IKBKG | Necroptosis |
| TLR3 | TLR3 | Necroptosis |
| TLR4 | TLR4 | Necroptosis |
| TRIF | TICAM1 | Necroptosis |
| ZBP1 | ZBP1 | Necroptosis |
| UPF1 | UPF1 | NMD |
| UPF2 | UPF2 | NMD |
| UPF3a | UPF3A | NMD |
| UPF3b | UPF3B | NMD |
| SMG1 | SMG1 | NMD |
| SMG5 | SMG5 | NMD |
| SMG6 | SMG6 | NMD |
| SMG7 | SMG7 | NMD |
| PABP | PABPC1 | NMD |
| PABPC4 | PABPC4 | NMD |
| PABPC3 | PABPC3 | NMD |
| eRF3a | GSPT1 | NMD |
| eRF3b | GSPT2 | NMD |
| eRF1 | ETF1 | NMD |
| PP2A | PPP2CA | NMD |
| SLBP | SLBP | NMD |
| PYM | PYM1 | NMD |
| SMG8 | SMG8 | NMD |
| SMG9 | SMG9 | NMD |
| POP2 | CNOT8 | NMD |
| XRN1 | XRN1 | NMD |
| CASC3 | CASC3 | NMD |
| Y14 | RBM8A | NMD |
| MAGOH | MAGOH | NMD |
| MAGOHB | MAGOHB | NMD |
| DDX5 | DDX5 | NMD |
| DDX17 | DDX17 | NMD |
| RUVBL1 | RUVBL1 | NMD |
| RUVBL2 | RUVBL2 | NMD |
| DHX34 | DHX34 | NMD |
| MOV10 | MOV10 | NMD |
| PNRC2 | PNRC2 | NMD |
| SMG10 | TTI1 | NMD |
| NBAS/NAG | NBAS | NMD |
| GNL2 | GNL2 | NMD |
| SEC13 | SEC13 | NMD |
| SEC31a | SEC31A | NMD |
| RNPS1 | RNPS1 | NMD |
| APOBEC1 | APOBEC1 | NMD |
| ACF | ACF | NMD |
| DCP2 | DCP2 | NMD |
| DCP1a | DCP1A | NMD |
| CBP80 | NCBP1 | NMD |
| CBP20 | NCBP2 | NMD |
| DNA-PK | PRKDC | NMD |
| ATM | ATM | NMD |
| ATR | ATR | NMD |
| TRRAP | TRRAP | NMD |
| CACNB4 | CACNB4 | NMD |
| CALHM3 | CALHM3 | NMD |
| CALHM2 | CALHM2 | NMD |
| CALHM1 | CALHM1 | NMD |
| BMP7 | BMP7 | NMD |
| GNB4 | GNB4 | NMD |
| TGIF1 | TGIF1 | NMD |
| SMAD1 | SMAD1 | NMD |
| STAU1 | STAU1 | NMD |
| STAU2 | STAU2 | NMD |
| FSD1L | FSD1L | NMD |
| TNRC5 | CNPY3 | NMD |
| PDRG1 | PDRG1 | NMD |
| TNRC1 | ZNF384 | NMD |
| 4EBP1 | EIF4EBP1 | NMD |
| PAIP1 | PAIP1 | NMD |
| PAIP2 | PAIP2 | NMD |
| eIF2A | EIF2S1 | Translation |
| eIF2S2 | EIF2S2 | Translation |
| eIF3D | EIF3D | Translation |
| eIF3K | EIF3K | Translation |
| eIF4E | EIF4E | Translation |
| eIF2S3 | EIF2S3 | Translation |
| eIF3E | EIF3E | Translation |
| eIF4G1 | EIF4G1 | Translation |
| eIF3B | EIF3B | Translation |
| eIF3A | EIF3A | Translation |
| eIF3G | EIF3G | Translation |
| eIF3F | EIF3F | Translation |
| eIF3I | EIF3I | Translation |
| eIF3H | EIF3H | Translation |
| eIF2AK2 | EIF2AK2 | Translation |
| eIF3M | EIF3M | Translation |
| eIF3C | EIF3C | Translation |
| eIF3CL | EIF3CL | Translation |
| eIF3J | EIF3J | Translation |
| eIF5A | EIF5A | Translation |
| eIF2AK1 | EIF2AK1 | Translation |
| eIF3L | EIF3L | Translation |
| eIF4B | EIF4B | Translation |
| eIF2S3B | EIF2S3B | Translation |
| eIF2AK3 | EIF2AK3 | Translation |
| eIF1AX | EIF1AX | Translation |
| eIF5 | EIF5 | Translation |
| eIF2AK4 | EIF2AK4 | Translation |
| eIF5A2 | EIF5A2 | Translation |
| eIF5AL1 | EIF5AL1 | Translation |
| eIF4A1 | EIF4A1 | Translation |
| eIF2B5 | EIF2B5 | Translation |
| eIF2B1 | EIF2B1 | Translation |
| eIF2B4 | EIF2B4 | Translation |
| eIF2B2 | EIF2B2 | Translation |
| eIF2A | EIF2A | Translation |
| eIF4A2 | EIF4A2 | Translation |
| eIF4EBP1 | EIF4EBP1 | Translation |
| eIF6 | EIF6 | Translation |
| eIF1AY | EIF1AY | Translation |
| eIF4G3 | EIF4G3 | Translation |
| MRI1 | MRI1 | Translation |
| eIF1 | EIF1 | Translation |
| eIF4H | EIF4H | Translation |
| eIF5B | EIF5B | Translation |
| eIF4EBP2 | EIF4EBP2 | Translation |
| eIF4G | EIF4G1 | Translation |
| eIF4G2 | EIF4G2 | Translation |
| eIF4G3 | EIF4G3 | Translation |
| eIF4A1 | EIF4A1 | Translation |
| eIF4A2 | EIF4A2 | Translation |
| eIF3 | EIF3A | Translation |
| eIF2A | EIF2A | Translation |
| eIF4A3 | EIF4A3 | Translation |
| eIF5A | EIF5A | Translation |
| eIF5AL1 | EIF5AL1 | Translation |
| eIF4G1 | EIF4G1 | Translation |
| eIF4E | EIF4E | Translation |
| eIF4B | EIF4B | Translation |
| eIF3H | EIF3H | Translation |
| eIF3F | EIF3F | Translation |
| eIF4A2 | EIF4A2 | Translation |
| eIF4A1 | EIF4A1 | Translation |
| eIF2AK3 | EIF2AK3 | Translation |
| eIF2B3 | EIF2B3 | Translation |
| eIF3I | EIF3I | Translation |
| eIF3L | EIF3L | Translation |
| eIF4E2 | EIF4E2 | Translation |
| eIF4E3 | EIF4E3 | Translation |
| eIF4EBP3 | EIF4EBP3 | Translation |
| PDK1 | PDK1 | mTOR |
| AKT | AKT1 | mTOR |
| AKT2 | AKT2 | mTOR |
| AKT3 | AKT3 | mTOR |
| TSC1 | TSC1 | mTOR |
| TSC2 | TSC2 | mTOR |
| TBC1D7 | TBC1D7 | mTOR |
| RHEB | RHEB | mTOR |
| RAPTOR | RPTOR | mTOR |
| Pras40 | AKT1S1 | mTOR |
| Deptor | DEPTOR | mTOR |
| MLST8 | MLST8 | mTOR |
| S6K1 | RPS6KB1 | mTOR |
| Tel2 | TELO2 | mTOR |
| FKBP1A | FKBP1A | mTOR |
| Rictor | RICTOR | mTOR |
| Protor2 | PRR5L | mTOR |
| Protor1 | PRR5 | mTOR |
| mSin1 | MAPKAP1 | mTOR |
| Rag A | RRAGA | mTOR |
| Rag B | RRAGB | mTOR |
| Rag C | RRAGC | mTOR |
| Rag D | RRAGD | mTOR |
| FLCN | FLCN | mTOR |
| FNIP1 | FNIP1 | mTOR |
| FNIP2 | FNIP2 | mTOR |
| RAGULATOR | LAMTOR1 | mTOR |
| RAGULATOR2 | LAMTOR2 | mTOR |
| RAGULATOR5 | LAMTOR5 | mTOR |
| RAGULATOR4 | LAMTOR4 | mTOR |
| RAGULATOR3 | LAMTOR3 | mTOR |
| SLC38A9 | SLC38A9 | mTOR |
| SAMTOR | BMT2 | mTOR |
| CASTOR3 | CASTOR3 | mTOR |
| CASTOR | CASZ1 | mTOR |
| DVL1 | DVL1 | mTOR |
| DVL2 | DVL2 | mTOR |
| DVL3 | DVL3 | mTOR |
| DEPDC5 | DEPDC5 | mTOR |
| NPRL2 | NPRL2 | mTOR |
| Mios | MIOS | mTOR |
| Sec13 | SEC13 | mTOR |
| Seh1l | SEH1L | mTOR |
| WDR24 | WDR24 | mTOR |
| WDR59 | WDR59 | mTOR |
| KPTN | KPTN | mTOR |
| ITFG2 | ITFG2 | mTOR |
| C12orf66 | C12orf66 | mTOR |
| SZT2 | SZT2 | mTOR |
| PI3K | PIK3CA | mTOR |
| PTEN | PTEN | mTOR |
| SGK1 | SGK1 | mTOR |
| PRKCa | PRKCA | mTOR |
| Mknk1 | MKNK1 | mTOR |
| COPB1 | COPB1 | Essential |
| COPS6 | COPS6 | Essential |
| Importin beta-1 | KPNB1 | Essential |
| Nup133 | NUP133 | Essential |
| RPB1 | POLR2A | Essential |
| RPB4 | POLR2D | Essential |
| PSMB2 | PSMB2 | Essential |
| PSMB3 | PSMB3 | Essential |
| RPL19 | RPL19 | Essential |
| RPL36 | RPL36 | Essential |
| Beta-defensin 129 | DEFB129 | Non-essential |
| DMRTB1 | DMRTB1 | Non-essential |
| MAGEB3 | MAGEB3 | Non-essential |
| NLRP5 | NLRP5 | Non-essential |
| Neuropeptide S receptor | NPSR1 | Non-essential |
| PIWIL3 | PIWIL3 | Non-essential |
| PLA2G2E | PLA2G2E | Non-essential |
| Sarcoma Antigen 1 | SAGE1 | Non-essential |
| SPATA16 | SPATA16 | Non-essential |
| TG6 | TGM6 | Non-essential |
